# Carnosine Activates Cellular Stress Response in Podocytes and Reduces Glycative and Lipoperoxidative Stress

**DOI:** 10.3390/biomedicines8060177

**Published:** 2020-06-26

**Authors:** Maria Scuto, Angela Trovato Salinaro, Sergio Modafferi, Alessandra Polimeni, Tilman Pfeffer, Tim Weigand, Vittorio Calabrese, Claus Peter Schmitt, Verena Peters

**Affiliations:** 1 Department of Biomedical and Biotechnological Sciences, University of Catania, 95124 Catania, Italy; mary-amir@hotmail.it (M.S.); Trovato@unict.it (A.T.S.); sergio.modafferi@gmail.com (S.M.); alessandrapolimeni@icloud.com (A.P.); 2 Centre for Pediatric and Adolescent Medicine, University of Heidelberg, 69117 Heidelberg, Germany; tilman.Pfeffer@med.uni-heidelberg.de (T.P.); tim.weigand@med.uni-heidelberg.de (T.W.); verena.peters@med.uni-heidelberg.de (V.P.)

**Keywords:** carnosine, glucose, diabetic nephropathy cellular stress response, oxidative stress, vitagenes

## Abstract

Carnosine improves diabetic complications, including diabetic nephropathy, in in vivo models. To further understand the underlying mechanism of nephroprotection, we studied the effect of carnosine under glucose-induced stress on cellular stress response proteins in murine immortalized podocytes, essential for glomerular function. High-glucose stress initiated stress response by increasing intracellular heat shock protein 70 (Hsp70), sirtuin-1 (Sirt-1), thioredoxin (Trx), glutamate-cysteine ligase (gamma-glutamyl cysteine synthetase; γ-GCS) and heme oxygenase-1 (HO-1) in podocytes by 30–50% compared to untreated cells. Carnosine (1 mM) also induced a corresponding upregulation of these intracellular stress markers, which was even more prominent compared to glucose for Hsp70 (21%), γ-GCS and HO-1 (13% and 20%, respectively; all *p* < 0.001). Co-incubation of carnosine (1 mM) and glucose (25 mM) induced further upregulation of Hsp70 (84%), Sirt-1 (52%), Trx (35%), γ-GCS (90%) and HO-1 (73%) concentrations compared to untreated cells (all *p* < 0.001). The glucose-induced increase in 4-hydroxy-*trans*-2-nonenal (HNE) and protein carbonylation was reduced dose-dependently by carnosine by more than 50% (*p* < 0.001). Although podocytes tolerated high carnosine concentrations (10 mM), high carnosine levels only slightly increased Trx and γ-GCS (10% and 19%, respectively, compared to controls; *p* < 0.001), but not Hsp70, Sirt-1 and HO-1 proteins (*p* not significant), and did not modify the glucose-induced oxidative stress response. In podocytes, carnosine induced cellular stress tolerance and resilience pathways and was highly effective in reducing high-glucose-induced glycative and lipoperoxidative stress. Carnosine in moderate concentrations exerted a direct podocyte molecular protective action.

## 1. Introduction

Carnosine (β-alanyl-L-histidine) and anserine (β-alanyl-N-methyl-L-histidine) are natural histidine-containing dipeptides (HDPs), commonly found in animal tissues that are excitable, but are absent in fungi, plants or other eukaryotes [1]. Skeletal muscles and the central nervous system represent tissues where these compounds are measured at significantly high concentrations (in the range of 0.6–30 mM) in humans and all vertebrates [2]. Recently, several in vivo studies demonstrated the potential of carnosine as an agent for the attenuation of different types of chronic diseases related to oxidative and glycative stress [3,4], such as diabetes-associated complications, in particular, diabetic nephropathy (DN) [5,6]. Moreover, carnosine has a role in the scavenging of carbonyls [5,6,7,8,9] and reactive oxygen species [10], and also inhibits glycation [11] and angiotensin-converting enzymes [12,13].

Podocytes play a role in preserving the integrity of glomerular function under normal conditions and are the target of many forms of physiological and pathophysiological stress. Podocyte injury leads to proteinuria and in patients with diabetes mellitus, to the development of DN [14,15,16]. Oxidative and glycative stress represents the starting point for the induction of markers of reactive oxygen species (ROS), advanced glycation end products (AGEs), protein carbonylation and lipid peroxidation adducts (HNE adducts) that are crucial to the initiation of apoptosis in podocytes [17]. Previous studies confirmed that ROS are overexpressed in podocytes in DN [18]. Podocytes have a prominent role in renal carnosine metabolism. However, the precise molecular mechanism of the protective action is not yet sufficiently understood. It has been discussed that carnosine may prevent apoptosis [19,20] and may upregulate defensive mechanisms such as heat shock proteins [21]. In addition, it seems to exhibit potent antiglycation properties [22]. In rats with streptozotocin-induced diabetes, carnosine treatment prevented apoptosis of glomerular cells and podocyte loss [23]. Previously, we have shown that carnosine and its methylated analog anserine exert protective actions in human renal tubular epithelial cells (HK-2) under diabetic conditions [24]. Podocytes and tubular and mesangial cells tolerate carnosine and anserine in high concentrations, but to different degrees. Carnosine was tolerated best by podocytes (EC_50_ = 9.1 mM), whereas the tolerance by tubular cells (EC_50_ = 4.1 mM) to carnosine was much lower [25]. The uptake of carnosine in podocytes is very low, although the putative carnosine transporter PHT1 is expressed in those cells [26].

While the protective effects of carnosine have been studied in tubular epithelial cells, respective data in podocytes are scant, despite the fact that podocytes play a key role in renal function and in contrast to tubular cells, have very limited capacity to regenerate, following injury. The cellular stress response pathway involves redox survival vitagenes, genes encoding for heat shock protein 70 (Hsp70), gamma-glutamyl cysteine synthetase (γ-GCS), heme oxygenase-1 (HO-1), as well as thioredoxin (Trx) and sirtuin-1 (Sirt-1) protein systems, which confer a cytoprotective state in a great variety of human diseases, particularly, in diabetic nephropathy [27,28,29,30,31]. We therefore investigated whether vitagenes are upregulated in murine podocytes in the presence of both normal and high glucose concentrations in response to increasing carnosine concentrations. We used two carnosine concentrations (1 mM and 10 mM), the latter being above the physiological level, to investigate the effects.

## 2. Materials and Methods

### 2.1. Cell Culture

Podocytes (immortalized podocytes of murine origin, ImmortoMouse, Charles River, Wilmington, MA, USA) were cultured in RPMI 1640 medium (Thermo Fisher Scientific, Waltham, MA, USA) containing 10% (*v*/*v*) inactivated fetal calf serum (Biochrom, Berlin, Germany), 1% streptomycin and penicillin (*v*/*v*) (Thermo Fisher Scientific, Waltham, MA, USA) and 10 U/μL γ-interferon (Roche Diagnostics, Mannheim, Germany) with 5% CO_2_ in flasks coated with collagen type I (BD Biosciences, Bedford, MA, USA) and maintained at 33 °C. To allow them to differentiate, cells were first detached with trypsin (Thermo Fisher Scientific, Waltham, MA, USA) and then seeded at 5000 cells/cm^2^ for 12–14 days, under 5% CO_2_ in growth medium at 33 °C in the absence of γ-interferon.

### 2.2. Western Immunoblotting

Podocytes were homogenized in 0.1 M NaCl, 0.01 M Tris-Cl (pH 7.6), 0.001 M EDTA (pH 8.0), 0.001 M PMSF and 1× protease inhibitor cocktail (Sigma, St. Louis, MO, USA). Proteins, determined with the bicinchoninic acid (BCA) method (Pierce Chemical, Dallas, TX, USA), were taken from each sample at a concentration of 50 μg and boiled for 3 min in a buffer containing 2.5% SDS, 40 mM Tris-HCl, 5% 2-mercaptoethanol, 0.025 mg/mL of bromophenol blue and 5% glycerol, before being separated by SDS-PAGE gels at 4–20% (Bio-Rad Laboratories, Hercules, CA, USA), which was run for 60 min at 100 V. Separated proteins were transferred to a nitrocellulose membrane (Bio-Rad Laboratories, Hercules, CA, USA) in a transfer buffer containing 192 mM glycine, 25 mM Tris, 0.05% SDS and 20% *v*/*v* methanol for 1 h at 100 V. Staining with Ponceau was used to confirm protein transfer to membranes. Membranes were then incubated for 1 h at room temperature (RT) in PBS and 0.1% Tween 20 (T-PBS) containing 2% milk powder and probed overnight (4 °C in T-PBS) with polyclonal antibodies specific for Trx, Sirt-1, Hsp70, HO-1 and γGCS protein and HNE-protein adduct (Santa Cruz Biotech. Inc., Dallas, TX, USA). Goat polyclonal antibody specific for β-actin (SC-1615, Santa Cruz Biotech. Inc., Dallas, TX, USA) was used for quantification. Unbound antibodies in excess were removed by washing and then incubated for 1 h at room temperature with the secondary polyclonal antibody coupled to horseradish peroxidase enzyme. SuperSignal detection system (Pierce Chemical, Dallas, TX, USA) was used as the luminescent substrate before analysis with the Molecular Imaging software and quantification (Bioscience, London, UK). Each experiment was performed in triplicate before statistical analysis.

### 2.3. Western Blot of Carbonylated Proteins

An OxyBlot^TM^ Protein Oxidation kit (Merck Millipore, Darmstadt, Germany) was used to analyze protein carbonyls. Briefly, denatured samples containing 15 μg protein were derivatized by adding 10 μL of 1× DNPH (2,4 dinitrophenolhydrazine) solution before incubation at room temperature for 15 min. After neutralization of protein samples, derivatized proteins were separated by SDS/PAGE. A primary antibody specific for DNPH was used and detected by luminescence (SuperSignal detection system kit; Pierce Chemical, Dallas, TX, USA). Bands were then quantified (Gel-Logic 2200-PRO Bioscience, London, UK) and analyzed (Image Lab™ Software, Version 6.0, Bio-Rad, Laboratories, Inc., U.S., Canada)

### 2.4. Statistical Analysis

Each experiment was performed in triplicate and data were given as mean and standard deviation (SD). Student’s *t*-test was used to compare groups. A *p*-value of <0.05 was considered to be significant. Significance in experiments comparing more than two groups was evaluated by one-way analysis of variance, followed by post hoc analysis using Tukey’s test.

## 3. Results

### 3.1. Effect of Carnosine in Glucose-Stressed Podocyte Cells

ROS, when generated at high levels, increases the expression of cytoprotective genes involved in detoxification reactions, the preservation of mitochondrial function and cell survival stress-responsive vitagenes [32].

**Figure 1:**
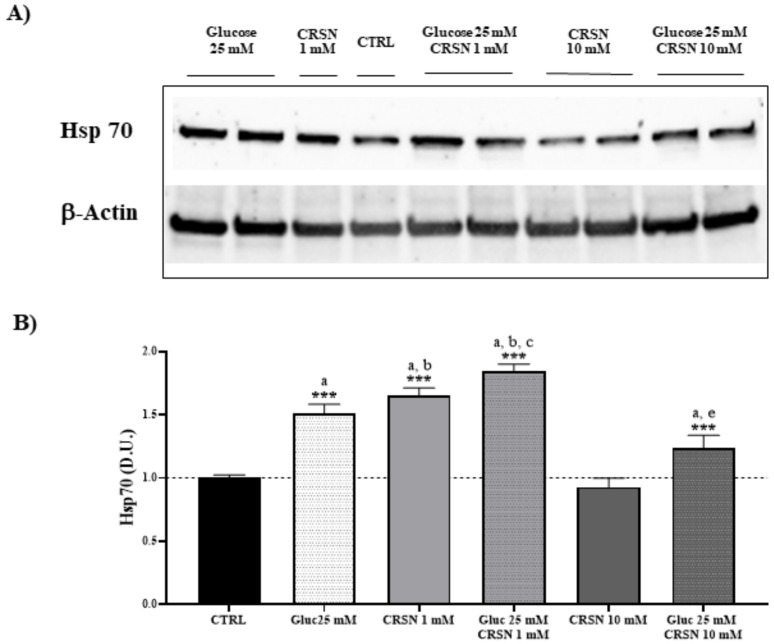
Cellular heat shock protein 70 (Hsp70) concentration (normalized to untreated cells) significantly increased in murine immortalized podocytes under glucose stress (25 mM; *p* = 3.7 × 10^−7^) or under carnosine addition (1 mM; *p* = 1.2 × 10^−8^). Co-incubation with carnosine (1 mM) and glucose (25 mM) induced further upregulation of Hsp70 protein concentrations compared to single treatment (*p* = 4.7 × 10^−3^ vs. glucose and *p* = 0.001 vs. carnosine) or control (*p* = 2.1 × 10^−9^), determined by western blotting (**A**) and quantified with an imaging software normalized to respective β-concentrations (**B**). Note: *n* = 5, a = significant increase vs. control, b = significant increase vs. glucose (25 mM), c = significant increase vs. carnosine (1 mM), d = significant increase vs. glucose (25 mM) + carnosine (10 mM), e = significant decreased vs. glucose (25 mM), *** *p* < 0.001.

To investigate the effect of carnosine antioxidant capacity to modulate the vitagene pathway, we performed experiments in which the levels of the inducible isoform Hsp70 were evaluated by western blot analysis in SV-40 immortalized murine podocytes in the presence of both high glucose (25 mM for 24 h) and normal glucose (11 mM). Glucose-stressed podocytes increased Hsp70 protein concentration by 30–50% compared to untreated cells (Figure 1, Table 1; *p* < 0.001) and normalized to respective β-actin concentrations. As reported in Figure 1, incubation with carnosine (1 mM) alone also induced a corresponding upregulation of the intracellular stress marker Hsp70, which was 21% more prominent compared to glucose alone. Co-incubation with carnosine (1 mM) and glucose (25 mM) induced further upregulation of Hsp70 protein by 84% as compared to untreated cells (*p* < 0.001). Co-incubation with carnosine (1 mM) and glucose (25 mM) increased Hsp70 concentrations by 21% compared to glucose alone (*p* < 0.01). The presence of carnosine alone at a high concentration (10 mM) did not increase Hsp70 protein expression. Moreover, co-incubation with a high concentration of carnosine (10 mM) increased the concentration of the inducible isoform Hsp70 by 24% (*p* < 0.001 vs. control), whereas only a slight reduction (about 8%) of Hsp70 expression was observed compared to glucose alone (Table 1; *p* < 0.05).

**Figure 2:**
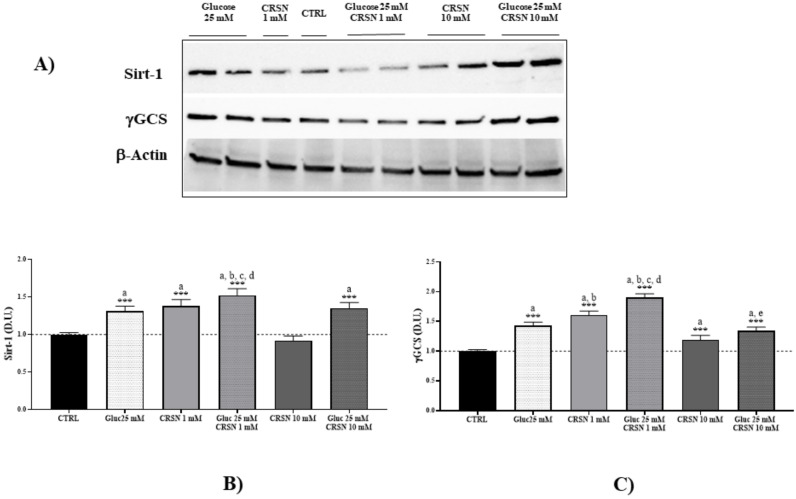
Cellular sirtuin-1 (Sirt-1) and gamma-glutamyl cysteine synthetase (γ-GCS) protein concentrations (normalized to untreated cells) significantly increased in murine immortalized podocytes under glucose stress (25 mM; *p* = 1.4 × 10^−3^ and *p* = 5.5 × 10^−7^, respectively) or under carnosine addition (1 mM; *p* = 1.0 × 10^−3^ and *p* = 4.4 × 10^−8^, respectively). Co-incubation with carnosine (1 mM) and glucose (25 mM) induced further upregulation of Sirt-1 and γ-GCS proteins compared to single treatment (*p* = 0.002 vs. glucose and *p* = 0.03 vs. carnosine for Sirt-1; *p* = 1.6 × 10^−6^ vs. glucose and *p* = 6.7 × 10^−3^ vs. carnosine for γ-GCS) or control (*p* = 1.1 × 10^−6^ and *p* = 1.1 × 10^−8^ for Sirt-1 and γ-GCS, respectively), determined by western blotting (**A**) and quantified with an imaging software normalized to respective β-actin concentrations (**B**,**C**). Note: *n* = 5, a = significant increase vs. control, b = significant increase vs. glucose (25 mM), c = significant increase vs. carnosine (1 mM), d = significant increase vs. glucose (25 mM) + carnosine (10 mM), *** *p* < 0.001.

In response to incubation with 25 mM glucose, we found that Sirt-1 protein was upregulated by 30–50% compared to untreated cells (Figure 2A, Table 1; *p* < 0.001). Incubation with carnosine (1 mM) also induced a respective upregulation of Sirt-1 concentration (*p* < 0.001 vs. untreated cells). In addition, co-incubation with 1 mM carnosine and 25 mM glucose induced further upregulation of Sirt-1 by 52% (*p* < 0.001 vs. untreated control). Co-incubation with carnosine increased Sirt-1 expression by 16% compared to high glucose alone (*p* < 0.01). Carnosine alone at high concentrations (10 mM) only slightly increased Sirt-1 protein compared to controls (*p* < 0.05), but did not increase Sirt-1 protein concentration compared to glucose alone (*p* not significant). Co-incubation with 10 mM carnosine concentration increased Sirt-1 expression by 35% compared to untreated cells (*p* < 0.001), as reported in Figure 2B.

**Figure 3:**
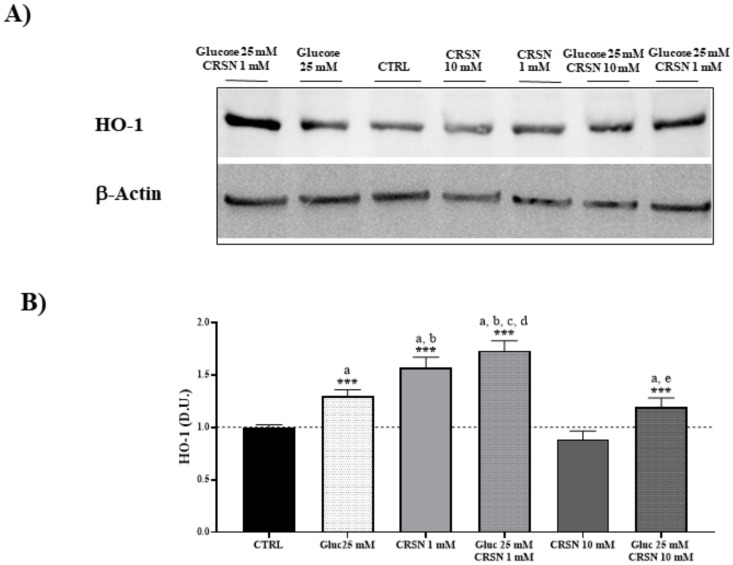
Cellular heme oxygenase-1 (HO-1) protein concentration (normalized to untreated cells) significantly increased in murine immortalized podocytes under glucose stress (25 mM; *p* = 5.4 × 10^−6^) or under carnosine addition (1 mM; *p* = 1.2 × 10^−6^). Co-incubation with carnosine (1 mM) and glucose (25 mM) induced further upregulation of HO-1 protein concentration compared to single treatment (*p* = 1.2 × 10^−6^ vs. glucose and *p* = 0.03 vs. carnosine) or control (*p* = 1.7 × 10^−7^), determined by western blotting (**A**) and quantified with an imaging software normalized to respective β-actin concentrations (**B**). Note: *n* = 5, a = significant increase vs. control; b = significant increase vs. glucose (25 mM), c = significant increase vs. carnosine (1 mM), d = significant increase vs. glucose (25 mM) + carnosine (10 mM), e = significant decrease vs. glucose (25 mM), *** *p* < 0.001.

Consistent with the notion that in response to environmental changes and other stressful conditions promoting proteotoxicity, cells adaptively activate the synthesis and accumulation of redox sensitive proteins, we measured HO-1 and γ-GCS, two proteins involved in redox homeostasis and in the synthesis of glutathione, respectively [33,34]. Under the same experimental conditions, we showed that glucose-stressed podocytes significantly increased γ-GCS and HO-1 expression by about 30–50% (Figure 2 and Figure 3, Table 1; *p* < 0.001 compared to controls). Incubation with 1 mM carnosine alone induced an upregulation of HO-1 and γ-GCS by 13% and 20%, respectively, compared to glucose treatment (*p* < 0.001). Notably, co-incubation with 1 mM carnosine and 25 mM glucose induced a further upregulation of HO-1 by 73% and of γ-GCS by 90% compared to untreated cells (each *p* < 0.001). Co-incubation with 1 mM carnosine increased HO-1 and γ-GCS both by 33% compared to high glucose alone (each *p* < 0.01). Treatment with 10 mM carnosine alone slightly increased γ-GCS protein concentration by 19% compared to controls (both *p* < 0.001), but did not affect HO-1 protein concentration (*p* not significant vs. untreated cells). Co-incubation with 10 mM carnosine increased HO-1 expression by 19% compared to controls (*p* < 0.01).

**Figure 4:**
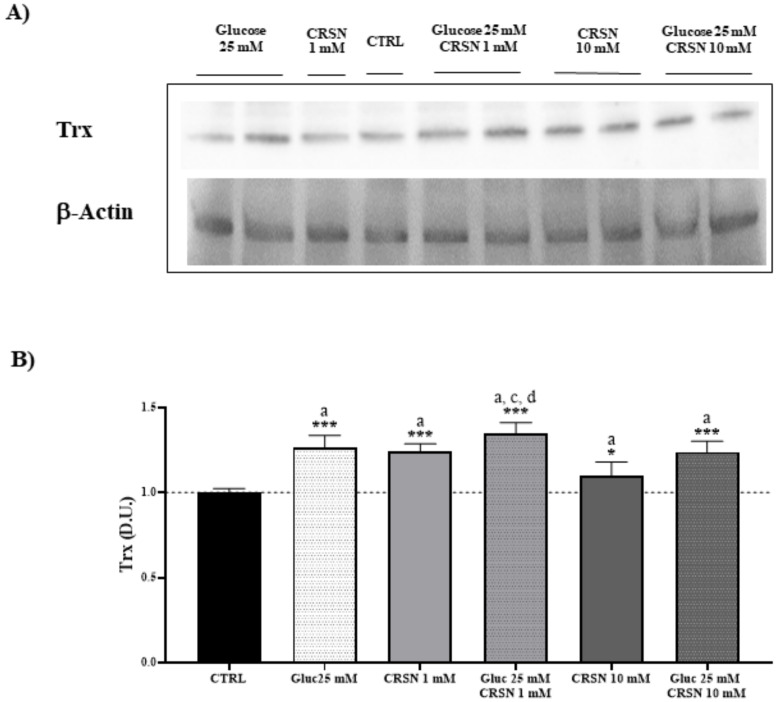
Cellular thioredoxin (Trx) protein concentration (normalized to untreated cells) significantly increased in murine immortalized podocytes under glucose stress (25 mM; *p* = 4.1 × 10^−3^) or under carnosine addition (1 mM; *p* = 5.2 × 10^−4^). Coincubation with carnosine (1 mM) and glucose (25 mM) induced further upregulation of Trx protein concentration compared to carnosine alone (*p* = 0.02) or control (*p* = 3.8 × 10^−6^), but not compared to glucose alone (*p* = 0.1), determined by western blotting (**A**) and quantified with an imaging software normalized to respective β-actin concentrations (**B**). Note: *n* = 5, a = significant increase vs. control, c = significant increase vs. carnosine (1 mM), d = significant increase vs. glucose (25 mM) + carnosine (10 mM), * *p* < 0.05, *** *p* < 0.001.

To shed more light on the implication that glucose-stressed podocytes modulate stress-responsive vitagenes, we measured redox-sensitive thioredoxin (Trx). In response to incubation with 25 mM glucose, Trx was upregulated by 30–50% compared to untreated cells (Figure 4, Table 1; *p* < 0.001). Incubation with carnosine (1 mM) also induced the upregulation of Trx protein expression compared to untreated cells (*p* < 0.001) and co-incubation with both glucose and carnosine induced an additional upregulation of Trx by 35% (*p* < 0.001). Compared to glucose treatment alone, Trx protein concentration was not elevated by co-incubation and was within the same range as glucose treatment. Moreover, carnosine in high levels (10 mM) increased Trx protein by only 10%, as compared to controls (*p* < 0.05). Co-incubation with the high carnosine concentration (10 mM) increased Trx expression by 24% compared to untreated cells (*p* < 0.001), but had no effect on Trx expression compared to glucose alone. 

### 3.2. Effect of Carnosine on Protein Carbonylation and Lipid Peroxidation (HNE) in Glucose-Stressed Podocytes

**Figure 5:**
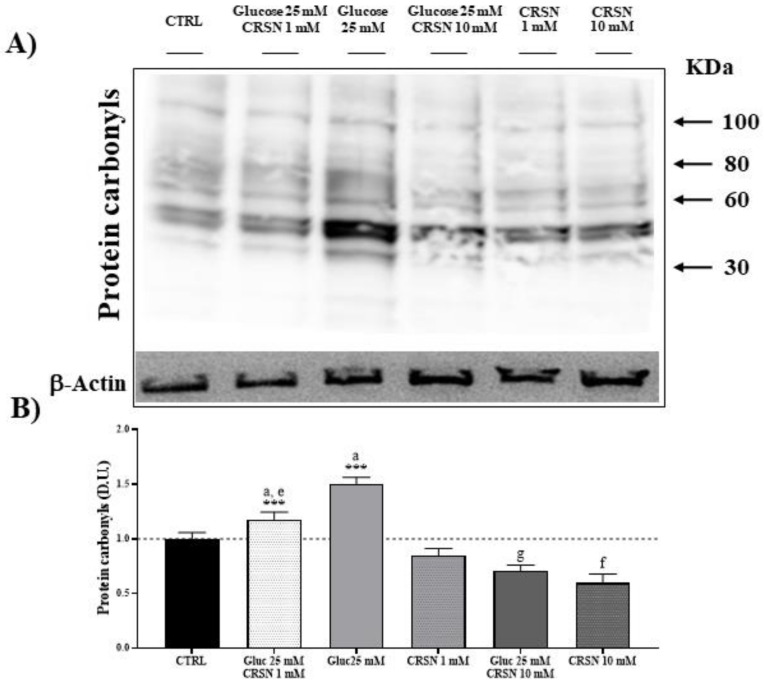
Glucose stress (25 mM) increased total protein carbonylation in murine podocytes, compared to controls by 50% (*p* = 1 × 10^−7^). Incubation with carnosine (1 mM and 10 mM) significantly reduced protein carbonylation (*p* = 0.007 and *p* = 3.0 × 10^−3^). Co-incubation with carnosine (1 mM and 10 mM) and glucose (25 mM) reduced protein carbonylation compared to glucose alone (*p* = 0.0001 and *p* = 5.4 × 10^−8^). Protein carbonylation was visualized by derivatization with 2,4-dinitrophenolhydrazine (DNPH) by western blotting (**A**) and quantified with an imaging software normalized to respective β-actin concentrations (**B**). Note: *n* = 5, a = increased vs. control, e = decreased vs. glucose (25 mM), f = decreased vs. carnosine (1 mM), g = decreased vs. glucose (25 mM) + carnosine (1 mM), *** *p* < 0.001 vs. control.

**Figure 6:**
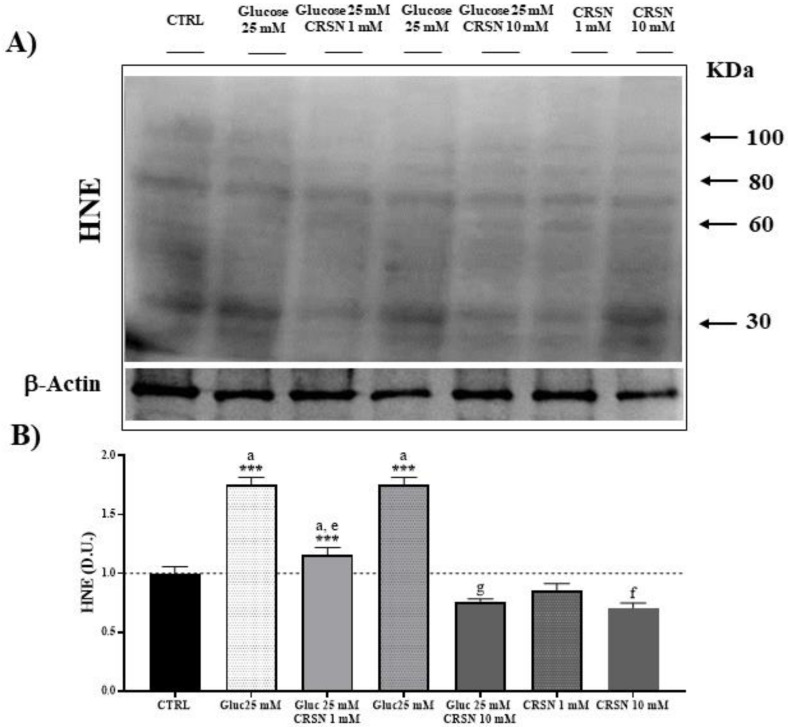
Glucose stress (25 mM) increased HNE (4-hydroxy-*trans*-2-nonenal) protein concentration in murine podocyte cells compared to controls by 75%. Incubation with carnosine (1 mM and 10 mM) significantly reduced HNE protein concentration (*p* = 0.007 and *p* = 3.1 × 10^−3^). Co-incubation with carnosine (1 mM and 10 mM) and glucose (25 mM) reduced HNE protein concentration vs. glucose alone (*p* = 1.1 × 10^−6^ and 1.9 × 10^−9,^ respectively), determined by western blotting (**A**) and quantified with an imaging software normalized to respective β-actin concentrations (**B**). Note: *n* = 5, a = increased vs. control, e = decreased vs. glucose (25 mM), f = decreased vs. carnosine (1 mM), g = decreased vs. glucose (25 mM) + carnosine (1 mM), *** *p* < 0.001 vs. control.

Oxidative stress represents a condition generally associated with the accumulation of products of protein oxidation, which are quantitated as protein carbonyls, as well as products of lipid peroxidation, determined as 4-hydroxynonenal (HNE) [35,36]. Carbonyl groups in amino acid moieties [35] and HNE derived from unsaturated fatty acids, as respective markers of proteotoxic and lipoperoxidative oxidant insult, are indicative of free-radical attacks and damage to proteins and lipids. Protein carbonylation and HNE, by binding via Michael addition to proteins, particularly at cysteine, histidine or lysine residues [37], affect cell homeostasis with deleterious consequences through accumulation of potentially toxic protein aggregates which result in cellular dysfunction. In podocytes exposed to glucose (25 mM), the level of protein carbonylation increased by about 50% and 4-hydroxynonenal (HNE) protein formation increased by about 75%, as compared to control (Table 2, Figure 5 and Figure 6; both *p* < 0.001). Incubation of the podocytes with carnosine decreased protein carbonylation dose-dependently by about 15% (1 mM carnosine) and about 40% (10 mM carnosine), and HNE formation was reduced by 15% (at 1 mM carnosine) and 29% (10 mM carnosine) compared to untreated cells. In glucose-stressed podocytes, the addition of 1 mM and 10 mM carnosine dose-dependently reduced protein carbonylation (by 20% at 1 mM and by 53% at 10 mM carnosine; all *p* < 0.001) compared to glucose alone. HNE formation was also dose-dependently reduced (by 34% at 1 mM and by 57% at 10 mM carnosine; all *p* < 0.001) compared to glucose alone.

## 4. Discussion

Diabetic nephropathy is a major complication in patients with type 1 or type 2 diabetes and represents the leading cause of end-stage renal disease. Accumulating evidence suggests that oxidative stress, defined as increased formation of reactive oxygen species (ROS) and/or decreased antioxidant potentials, plays a critical important role in the development of diabetic complications [32,38]. Specifically, reactive oxygen species (ROS) can oxidize proteins and membrane lipids following free-radical attacks, generating protein carbonylation and lipid hydroperoxides by binding via Michael addition, particularly at cysteine, histidine or lysine residues [37]. Oxidatively modified proteins are characterized by increased levels of protein-resident carbonyls, which is a measure of protein oxidation, or reactive alkenals bound to proteins, such as 4-hydroxy-2-nonenal, a consistent measure of lipoperoxidative insult. The most common oxidative post-translational modifications to a protein involve (a) carbonyl formation associated with homolytic radical scission of the primary peptide chain, as well as oxidation at side chain moieties of vulnerable amino acids and glycation end product formation as a result of a reaction between the protein and a reducing sugar or the lipid peroxidative product of a highly reactive alkenal; (b) covalent binding via Michael addition of 4-hydroxy-2-nonenal or other reactive alkenals reacting with Cys, Lys or His amino acid residues; and (c) nitration or nitrosylation of Tyr and Cys residues, respectively. Furthermore, Cys residues are especially vulnerable to free-radical-induced changes in cellular redox state and thus, thiol homeostasis exerts regulatory control of the activity of the proteome [39]. Notably, 4-hydroxy-*trans*-2-nonenal (HNE) is considered as an important marker of lipid peroxidation since it can accumulate in cells in relatively high concentrations and cause cell toxicity. The generation of potentially toxic protein aggregates via protein carbonylation induces cellular dysfunction as in the clinical settings of diabetic nephropathy, where oxidative stress underlies the pathophysiological accumulation of protein oxidation and lipid oxidation products, measured as protein carbonyls and HNE or ultraweak luminescence, respectively [35,36]. On the other hand, it is generally recognized that carnosine exerts a protective role in the context of DN [40]. In murine models of diabetes type 1 and 2, treatment with carnosine significantly decreased oxidative stress-induced increase of protein carbonyls and AGE formation, thus ameliorating glucose homeostasis and minimizing structural and functional renal dysfunction [26,41]. Whether the beneficial effects of carnosine result from interference with reactive metabolite-induced molecular pathomechanisms is yet unclear. Even though podocytes are essential in physiological glomerular homeostasis and minor alterations may result in severe glomerular barrier dysfunction, the molecular response to glucose stress and the action of carnosine have not yet been studied in detail. Therefore, we studied the effect of carnosine in glucose-stressed podocytes on cellular stress response proteins. Cellular stress markers are mediators of cytoprotection and tissue protection against a wide variety of injurious insults and are part of the integrated system for cellular stress tolerance and resilience [27,42]. The mechanism for glucotoxicity is, at least in part, mediated by overloads of reactive oxygen species.

We demonstrated that high-glucose stress initiates stress response by increasing cellular stress protein concentrations of Hsp70, Sirt-1, Trx, γ-GCS and HO-1 in podocytes, all part of the integrated system for cellular stress tolerance [29]. Upregulation of Hsp70, Sirt-1, Trx and HO-1 under high glucose in human tubular cells (HK-2 cells) has been previously shown [24]. Further, our data showed that carnosine (1 mM and 10 mM) severely reduced glucose-induced protein carbonylation and formation of HNE and suggested an important role of carnosine in the upregulation of Hsp70, Sirt-1, Trx, γ-GCS and HO-1 in untreated and glucose-stressed podocytes. This carnosine-induced upregulation has only been observed for 1 mM carnosine and was almost abolished at carnosine levels (10 mM) above the physiological range. This result can be explained with the emerging concept of hormesis. Vitagenes, encoding for the Hsp70, heme oxygenase, thioredoxin and sirtuin protein systems are targets of dietary antioxidants, such as carnosine, carnitines or polyphenols, which have recently been demonstrated to be neuroprotective through the activation of hormetic pathways [43]. Hormesis, cellular stress response and vitagenes are major critical determinants in aging and longevity, as well as in many biological conditions associated with redox-dependent mitochondrial alterations, including diabetes. The quantitative features of the hormetic dose response are similar across biological models and endpoints. This remarkable general feature suggests that the hormetic dose response, showing a biphasic profile of protection at lower dosages and abolishment of the effect or even toxicity at higher doses, may represent the first comprehensively based quantitative estimation of biological plasticity. For instance, downregulation of heat shock factor (HSF) reduces the lifespan and accelerates the formation of protein aggregates in *Caenorhabditis elegans* carrying mutations in different components of the IGF1R-mediated pathway, while, conversely, inhibition of IGF1R signaling results in HSF activation and promotes longevity by maintaining proteostasis [44], as also verified by our laboratory, in a *C. elegans* model of Parkinson’s disease treated with natural polyphenol antioxidants derived from olive oil [45,46]. Thus, regulation of endogenous oxidant/antioxidant balance via cellular stress response signaling represents a novel target for innovative approaches and therapeutic interventions in chronic damage associated with oxidant disorders [47]. Heat shock protein 70, whose expression is generally increased during stressful conditions, is a product of vitagenes involved in the defense against oxidative stress-derived proteotoxic insults [48], where it has been demonstrated to efficiently confer cytoprotection [49]. Hsp70 synthesis was found to be significantly increased in the serum of patients with type 2 diabetes, as compared to controls [50]. An increase in Hsp70 has been associated with decreased apoptosis, as evidenced by increased expression of Bcl-2 anti-apoptotic protein, reduced matrix metalloproteinases and blocked monocyte/microglial activation [33]. In tubular cells (human), anserine, not carnosine, increased intracellular Hsp70, as determined at both mRNA and protein levels [24], which indicates different cellular defense strategies in those cells. Here, we provide evidence that carnosine (and not only anserine, the methylated form of carnosine) strongly activates an array of cellular stress response and resilience systems and markedly reduces intracellular glycative and lipoperoxidative stress in podocytes.

Carnosine also activates the intracellular defense by activating Sirt-1 expression. Within the vitagene stress response pathway, sirtuin-1 protein (Sirt-1) has emerged with considerable interest. Sirt-1 is a conserved NAD^+^-dependent deacetylase involved in the control of histones and in maintaining resilience and survival to cellular stress [31]. Upregulation of sirtuin-1, a protein deacetylase, has been described to attenuate DN in different experiments reproducing diabetes conditions, as well as in mesangial cells, proximal tubular cells and podocytes [51]. It is known that sirtuin-1 is actively involved in the maintenance of cellular integrity and hence, the survival of podocytes. Consistent with this notion, a “proximal tubule-podocyte communication” has been inferred [52] by which tubular sirtuin-1 is able to downregulate claudin-1 expression in podocytes and thus afford protection against diabetes-induced albuminuria.

Glutathione (GSH) regulates redox-dependent cell signaling and reduced synthesis contributes to glutathione deficiency in patients with type 2 diabetes, and it is more marked in those with diabetic complications [53]. Glutathione is synthesized by the consecutive action of glutamate-cysteine ligase (a rate-limiting enzyme, also known as gamma-glutamyl cysteine synthetase; γ-GCS) and glutathione synthetase. We demonstrated that in podocytes, carnosine-mediated upregulation of γ-GCS was more prominent compared to glucose-mediated upregulation. In contrast, Catherwood and colleagues [54] reported reduced levels of GSH under high glucose compared with those grown in normal glucose concentrations, accompanied by decreased gene expression of both subunits of γ-GCS.

Carnosine also mediated the upregulation of HO-1 protein concentration in podocytes, which has also been reported in tubular cells [24]. HO-1, also referred to as Hsp32, belongs to the Hsp family and exerts an important role to catalyze the conversion of heme to biliverdin, ferrous iron and carbon monoxide, which can directly scavenge free radicals [55]. Heme oxygenases are dynamic sensors of cell oxidative stress and modulators of redox homeostasis [47]. HO-1 gene expression, which is controlled by factors such as pro-oxidant states or inflammation, is regulated mainly by two upstream enhancers, E1 and E2 [32]. Dong et al. [56] proposed that enhancing autophagy through increased HO-1 expression could be a potential therapeutic strategy in DN.

Trx was upregulated by high glucose or carnosine, but co-incubation showed no further upregulation of Trx compared to single treatment. Thioredoxins are proteins that act as antioxidants by facilitating the reduction of other proteins by cysteine thiol-disulfide exchange.

In conclusion, we provide evidence that carnosine activates the intracellular defense systems in podocytes and markedly reduces glycative and lipoperoxidative stress. Since podocytes are crucial for physiological glomerular barrier function and have a limited ability to repair and/or regenerate and the extent of podocyte loss is a major prognostic determinant in DN and other common renal diseases, upregulation of podocyte defense mechanisms by carnosine is a highly promising and yet underexplored therapeutic approach.

## Figures and Tables

**Table 1:** Cellular stress response protein concentration. Cellular heat shock protein 70 (Hsp70), sirtuin-1 (Sirt-1), thioredoxin (Trx), gamma-glutamyl cysteine synthetase (γ-GCS) and heme oxygenase-1 (HO-1) protein concentrations in podocytes treated with high glucose and carnosine (1 or 10 mM) alone or in combination. The derivatized proteins were separated by SDS/PAGE (see Figure 1, Figure 2, Figure 3 and Figure 4), quantified and normalized to untreated cells.

Stress Response Proteins	Hsp70	Sirt-1	Trx	γ-GCS	HO-1
Glucose (25 mM)	1.51 ± 0.07 ^a^	1.31 ± 0.07 ^a^	1.27 ± 0.07 ^a^	1.42 ± 0.06 ^a^	1.30 ± 0.06 ^a^
Carnosine(1 mM)	1.65 ± 0.06 ^a,b^	1.38 ± 0.08 ^a^	1.24 ± 0.04 ^a^	1.61 ± 0.06 ^a,b^	1.57 ± 0.10 ^a,b^
Glucose (25 mM) + Carnosine (1 mM)	1.84 ± 0.06 ^a,b,c,d^	1.52 ± 0.09 ^a,b,c,d^	1.35 ± 0.07 ^a,c,d^	1.90 ± 0.06 ^a,b,c,d^	1.73 ± 0.10 ^a,b,c,d^
Carnosine (10 mM)	0.93 ± 0.07	0.92 ± 0.06	1.10 ± 0.08 ^a^	1.19 ± 0.07 ^a^	0.89 ± 0.08
Glucose (25 mM) + Carnosine (10 mM)	1.24 ± 0.10 ^a,e^	1.35 ± 0.08 ^a^	1.24 ± 0.06 ^a^	1.34 ± 0.06 ^a^	1.19 ± 0.09 ^a,e^

Note: ^a^ = increased vs. control, ^b^ = increased vs. glucose (25 mM), ^c^ = increased vs. 1 mM carnosine, ^d^ = increased vs. glucose (25 mM) + carnosine (10 mM), ^e^ = decreased vs. glucose (25 mM).

**Table 2:** Oxidative stress-induced protein carbonylation and 4-hydroxynonenal (HNE) formation. Carnosine reduced glucose-induced protein carbonylation and formation of HNE. The derivatized proteins were separated by SDS/PAGE (Figure 5 and Figure 6), quantified and normalized to untreated cells.

Stress-Induced Protein Modification	Protein Carbonylation	HNE Formation
Glucose (25 mM)	1.5 ± 0.07 ^a^	1.75 ± 0.07 ^a^
Carnosine (1 mM)	0.85 ± 0.07	0.85 ± 0.07
Glucose (25 mM) + Carnosine (1 mM)	1.18 ± 0.07 ^a,e^	1.15 ± 0.07 ^a,e^
Carnosine (10 mM)	0.6 ±0.08 ^f^	0.71 ± 0.05 ^f^
Glucose (25 mM) + Carnosine (10 mM)	0.71 ± 0.06 ^g^	0.76 ± 0.02 ^g^

Note: ^a^ = increased vs. control, ^e^ = decreased vs. glucose (25 mM), ^f^ = decreased vs. carnosine (1 mM), ^g^ = decreased vs. glucose (25 mM) + carnosine (1 mM).
